# Editorial: Electric stimulation in the eye and brain: advancements and applications

**DOI:** 10.3389/fcell.2025.1643111

**Published:** 2025-07-02

**Authors:** Daisy Y. Shu, Yukari Nakano, Kin-Sang Cho, Anton Lennikov

**Affiliations:** ^1^ Faculty of Medicine and Health, School of Optometry and Vision Science, University of New South Wales (UNSW), Sydney, NSW, Australia; ^2^ R&D Div., R&D Center, Artificial Vision Institute, Nidek Co., Ltd., Gamagori, Aichi, Japan; ^3^ Department of Ophthalmology, Harvard Medical School, Schepens Eye Research Institute of Massachusetts Eye and Ear, Boston, MA, United States

**Keywords:** electrical stimulation (ES), retinal degeneration, neuroprotection, retinal ganglion cells (RGCs), transcorneal electrical stimulation (tcES), neural plasticity, retinal prosthetics, transcranial direct current stimulation (tDCS)

## Introduction

Electrical stimulation (ES), once regarded as a niche or experimental intervention, has rapidly emerged as a versatile and effective therapeutic approach for modulating, preserving, and restoring neural function in both the eye and brain at clinical (Schatz et al., 2017; Miura et al., 2023; Schatz et al., 2011) and preclinical levels (Enayati et al., 2020; Enayati et al., 2024; Gonzalez Calle et al., 2023). The origins of ES date back to early explorations of neurophysiological excitability; however, it is only in recent decades, with the advent of advanced bioengineering, neuroimaging, and molecular techniques, that ES has gained traction as a viable therapeutic modality. This Research Topic features eight articles, including six original research articles, one review, and one case report, that collectively illustrate the broad therapeutic potential and the mechanistic insights of ES. These contributions reinforce the concept of the eye as a window to the brain, offering a unique platform to explore the mechanistic and clinical impact of ES across multiple dimensions—from neuroprotection and prosthetic restoration to cellular reprogramming and neurological rehabilitation.

## From restoration to protection: reframing the role of ES in retinal degeneration

Historically, ES in ophthalmology has been predominantly associated with retinal prosthetics that aim to restore vision through direct stimulation of retinal neurons. However, the work by Yoo et al. broadened this paradigm by introducing the concept of modulation efficiency ratio (MER), a novel metric that compares the responsiveness of retinal ganglion cells (RGCs) in healthy versus degenerated primate retinas. Their findings illustrate that pathological hyperactivity in diseased retinal tissue significantly reduces RGC responsiveness to ES. This critical insight underscores the necessity of developing adaptive stimulation strategies that are specifically tailored to the altered biophysical environment of diseased tissues, marking a pivotal shift from a solely restorative focus to proactive neuroprotective strategies.

Azrad Leibovitch et al. further advanced the field by introducing a novel RCS rat model expressing the genetically encoded calcium indicator (GCaMP6f). This innovative model enables high-resolution, artifact-free optical monitoring of RGC activity in response to subretinal ES. This approach addresses longstanding challenges in electrophysiology, enabling high-resolution, artifact-free monitoring of retinal activity. This also offers a robust platform for tracking stimulus-response dynamics throughout retinal degeneration and guiding the refinement of therapeutic strategies.

However, the therapeutic potential of ES extends beyond prosthetic restoration alone. Gunes et al. demonstrated the neuroprotective effects of noninvasive transpalpebral ES in a *Rho* ^*−/−*^ mouse model of retinitis pigmentosa, showing significant improvements in cone survival and preserved visual function. Their work provides robust preclinical evidence that ES can act as a protective agent and mitigate retinal degeneration. Indeed, ES is not merely a restorative approach: it is capable of slowing degenerative processes.

Building on the theme of noninvasive stimulation, Morimoto contributed with a comprehensive review of transcorneal electrical stimulation (TES), a clinically accessible approach that applies weak currents via corneal electrodes to stimulate the inner retina. TES can stimulate RGCs without activating photoreceptors, making it a useful method for evaluating inner retinal function. In addition to its role in functional assessment, TES has been shown to exert neuroprotective effects on both RGCs and photoreceptors. The review delves into the underlying mechanisms, such as the upregulation of neurotrophic factors (e.g., IGF-1, BDNF, CNTF), the modulation of inflammatory pathways, and the activation of regenerative signaling cascades such as STAT3 and NF-κB. Importantly, Morimoto highlights the involvement of Müller glia and microglia as mediators of ES-induced tissue repair.

## Engineering better interfaces: toward integration and biocompatibility

Effective therapeutic ES demands not only precision in waveform and dosage, but also stable and effective interfaces with the neural substrate. To address this critical need, Shpun et al. presented compelling data on how biomimetic surface modifications, using integrin-targeted peptides, such as RGD and YIGSR, significantly enhance the adhesion of retinal cells to gold electrode surfaces. Their interdisciplinary study bridged material science and cellular biology, establishing foundational design principles for next-generation neuroelectronic devices that prioritize both electrical performance and biocompatibility.

Similarly, Abbott et al. presented a minimally invasive, chronically implantable suprachoroidal device engineered for long-term neuroprotective stimulation. Their rigorous preclinical safety assessment in feline models confirmed not only biotolerance, but also positional stability and a minimal inflammatory response, all of which are critical prerequisites for successful clinical application.

Together, these bioengineering-focused studies demonstrate that successful ES therapy is inseparable from the interface, where electrical signals interact with biological tissues. Better adhesion, precise localization of current delivery, and demonstrated long-term safety will dramatically increase the translational viability of ES.

## Neuroplasticity and rehabilitation: expanding beyond the retina

The therapeutic scope of ES is not limited to the retina. Indeed, two studies in this Research Topic extend the therapeutic promise of ES into the domain of post-stroke visual rehabilitation, involving re-engagement of cortical plasticity.

Diana et al. conducted a controlled study in which they used transcranial direct current stimulation (tDCS) applied to the occipital and parietal regions of individuals with homonymous visual field defects. Their results demonstrate improved visual search times following stimulation, with effects modulated by lesion characteristics and lateralization. Lian et al. further supported this concept with an insightful case report showing significant functional recovery following combined visual training and occipital tDCS in patients with cortical and optic nerve injuries.

Both studies underscore the synergistic potential of pairing ES with behavioral therapy, achieving enhanced visual performance and facilitating large-scale reorganization of the neural networks. Crucially, these cortical stimulation studies mirror the common themes emerging from retinal ES research: whether at the level of the retina or the cortex, ES can engage endogenous repair pathways, rewire surviving circuits, and restore meaningful function.

## Toward a unified framework for electric stimulation therapy

Taken together, the studies in this Research Topic advance a compelling new framework: ES as a versatile, systems-level therapy for neurodegeneration, circuit dysfunction, and tissue repair across retinal and cortical networks. What was once considered speculative is now emerging as a promising clinical therapy, propelled forward by rigorous experimentation, innovative models, and advancements in device engineering. We are witnessing the evolution of an interdisciplinary field that integrates bioelectric mechanisms, neurobiology, materials science, and rehabilitative medicine into a coherent therapeutic framework.

This Research Topic stands as a testament to the transformative power of electricity, not only to stimulate but also as a therapeutic tool to protect neural tissues, restore lost function, and heal.
